# CAR-T cell therapies in autoimmune rheumatic diseases: a brief report on the clinical trial landscape, current status, and future perspectives

**DOI:** 10.3389/fimmu.2025.1630569

**Published:** 2025-10-22

**Authors:** Xiao Xu, Su-Hua Su

**Affiliations:** ^1^ Research Center of Molecular Medicine (Ph.D. Lab), Faculty of Nursing, Nantong Health College of Jiangsu Province, Nantong, China; ^2^ Department of Rheumatology, The First Affiliated Hospital of Zhejiang Chinese Medical University (Zhejiang Provincial Hospital of Chinese Medicine), Hangzhou, China

**Keywords:** CAR-T, autoimmune rheumatic diseases, clinical trial landscape, CAR-T therapy, cell therapy

## Abstract

Autoimmune rheumatic diseases (ARDs) are chronic inflammatory disorders where B cells play a key role. Traditional B-cell-targeted therapies have limitations, whereas CAR-T-cell therapy, which aims for a broader reset of the B-cell compartment by targeting B-cell surface markers such as CD19 or B-cell maturation antigen (BCMA), has unique advantages. Currently, most CAR-T cell trials for ARDs are in the early stages, with 64.29% (36/56 trials) of studies being phase I trials and only 7.14% (4/56 trials) progressing to phase II trials, primarily focusing on conditions, such as systemic lupus erythematosus (SLE) and lupus nephritis (LN). Geographically, clinical research is predominantly led by China (48% of trials [27/56 trials]) and the United States (34% of trials [19/56 trials]), although large-scale global collaborations remain limited, with only 3.6% (2/56 trials) of projects involving both U.S. and Chinese teams. Funding for these studies is driven primarily by non-leading pharmaceutical firms (75% [42/56 trials] of sponsors). Despite promising efficacy, e.g., CD19-targeted CAR-T cell therapy has induced significant clinical remission in refractory SLE patients, challenges remain, including high costs, complex production, and safety risks. Future progress requires expanding trials, optimizing CAR constructs, enhancing collaboration, and establishing safety monitoring networks, to promote the application of CAR-T cell therapy in ARDs and advance precision medicine.

## 1 Introduction

Autoimmune rheumatic diseases (ARDs) represent a group of chronic inflammatory disorders characterized by autoreactive antibodies, encompassing anti-synthetase syndrome (ASS), systemic sclerosis (SSc), systemic lupus erythematosus (SLE), rheumatoid arthritis (RA), antineutrophil cytoplasmic antibody-associated vasculitis (AAV), and primary Sjögren syndrome (pSS) (1). Globally, ARDs affect an estimated 3%-5% of the population (2). Disease-related pain and disability contribute to work productivity loss and diminished quality of life, whereas substantial treatment costs impose a significant socioeconomic burden (3). B cells play a pivotal role in ARDs pathogenesis through antigen presentation, T-cell activation, proinflammatory cytokine production, and the generation of circulating immune complexes (4). Although biologics that target CD20 on B cells initially offered therapeutic promise, their clinical utility remains constrained by limitations in real-world efficacy.

CD20-targeting monoclonal antibodies (e.g., rituximab) have heterogeneous therapeutic effects on systemic inflammatory diseases such as SLE and SSc (5–7). Notably, analysis of two pivotal multicenter RCTs (EXPLORER and LUNAR) revealed that rituximab failed to meet predefined primary efficacy endpoints in SLE management. This phenomenon may stem from efficient depletion of circulating B cells via effector cells (e.g., monocytes/macrophages), whereas CD19^+^ autoreactive plasmablasts persist in lymph nodes and bone marrow owing to limited antibody penetration and reduced effector cell density (e.g., natural killer (NK) cells) within these niches. Second-generation anti-CD20 antibodies (e.g., ocrelizumab^®^, obinutuzumab^®^) achieve deeper B-cell depletion but concomitantly increase the risk of opportunistic infections through potent immunosuppression (8).

Chimeric antigen receptor (CAR)-T cell therapy employs genetically reprogrammed T cells to eliminate pathogenic B cells via the targeting of surface markers (CD19 or B-cell maturation antigen (BCMA)), thereby restoring immune homeostasis (9). The CAR structure comprises an antigen-binding domain for target recognition, a transmembrane anchoring region, and intracellular signaling domains for T-cell activation. The therapeutic protocol involves T-cell harvesting, activation, genetic modification, ex vivo expansion, and reinfusion, enabling precise eradication of antigen-expressing cells to rectify immune dysregulation.

Current evidence highlights the unique advantages of CAR-T cell therapy: 1) direct cytolytic activity independent of exogenous effector cells and 2) tissue-homing capacity facilitating elimination of pathogenic B-cell subsets in antibody-impermeable anatomical sites (e.g., lymphoid follicles). Ohno et al. (10) demonstrated that CD19 CAR-T cells achieved complete depletion of CD19^+^/CD20^+^ B cells in lymph nodes while disrupting follicular architecture and follicular dendritic cell (FDC) networks in ARDs patients. Remarkably, CD19 CART cell therapy also eradicated tissue-infiltrating B cells in nonlymphoid organs, including the colon, kidney, and gallbladder.

These findings suggest that T-cell-based precision immunomodulation may overcome the therapeutic limitations of current B-cell-targeting approaches, particularly for ARDs patients with end-organ damage from autoreactive B cells (e.g., lupus nephritis, SSc-associated pulmonary fibrosis, glandular fibrosis in Sjögren’s syndrome, and myopathic atrophy in inflammatory myositis).

Since the 2017 FDA approval of tisagenlecleucel^®^ (first-in-class CD19-CAR T cell therapy for refractory acute lymphoblastic leukemia) (11), six CD19-CAR T cell products have been licensed for the treatment of B-cell malignancies and plasma cell dyscrasias (12, 13). Intriguingly, this B-cell-depleting modality has shown transformative potential in early-phase ARDs trials. The Universitätsklinikum Erlangen group first reported in 2021 that autologous CD19 CAR-T cell therapy induced sustained drug-free remission (≥18 months) in refractory SLE patients (14). Subsequent applications have expanded to SSc, dermatomyositis, and primary Sjögren’s syndrome. However, current trials are limited by small sample sizes (6–75 participants) and single-center designs, generating insufficient evidence for conventional regulatory approval in rheumatology. Notably, the FDA’s breakthrough therapy designation, which was originally established for oncology, may facilitate accelerated clinical translation of CAR T-cell therapies in ARDs through expedited review pathways.

Importantly, the scope of this analysis focused on CAR-T-cell therapies designed for broad depletion of B-lineage cells (e.g., via targets such as CD19 or BCMA) to achieve a reset of the immune system. While other innovative antigen-specific cytotoxic strategies, such as chimeric autoantibody receptor T (CAAR-T) cells, which eliminate only autoantibody-producing B cells with high specificity, and CAR-NK (natural killer) cell therapies, are emerging, these approaches involve distinct technological pathways.

This study systematically analyzes the clinical trial landscape of ARD therapeutics, identifies critical gaps in current strategies, and evaluates the translational potential of B-cell-resetting CAR-T-cell therapy on the basis of recent advancements. Our findings can inform the optimization of precision medicine paradigms for ARDs management and guide future research directions.

## 2 Materials and methods

By querying the Trialtrove database, which is owned by Citeline Clinical Intelligence (https://clinicalintelligence.citeline.com/trials/results), we conducted a comprehensive exploration and analysis of the clinical trial landscape for CART therapy for ARDs, with the search set to May 10, 2025. The search terms included ‘Drug disease: Lupus Nephritis, Systemic Lupus Erythematosus, Anti-Synthetase Syndrome, Sjögren’s Syndrome, Dermatomyositis, Scleroderma, Rheumatoid Arthritis, ANCA Associated Vasculitis, Myositis’, ‘Drug disease group: Immunological’, and ‘MeSH term ID: D008180, D001327, D012859, D003882, D012595, D001172, D014657, D009135’. On the basis of the classification criteria within the Trialtrove database and the inclusion criteria of clinical trials, SLE with and without nephritis is classified and defined as “systemic lupus erythematosus” and “lupus nephritis”, respectively. Systemic lupus erythematosus was diagnosed according to the 2019 EULAR/ACR criteria (15). The classification of SLE requires the presence of a positive antinuclear antibody (ANA) test as an entry criterion. The additive criteria consisted of seven clinical (i.e., constitutional, hematologic, neuropsychiatric, mucocutaneous, serosal, musculoskeletal, and renal) and three immunologic (i.e., antiphospholipid antibodies [aPLs], complement proteins, and SLE-specific antibodies) categories, each of which were weighted from 2-10. Patients are classified as having SLE with a score of 10 or more points (15). In the diagnosis and classification of lupus nephritis (LN), the diagnostic criteria for systemic lupus erythematosus (SLE) must first be met. Moreover, the diagnosis of LN is ideally confirmed by a kidney biopsy (16). We generally perform a kidney biopsy in patients who have one or more of the following clinical manifestations: **1).** Urine protein excretion greater than 500 mg/day; **2).** An active urinary sediment with persistent hematuria (five or more red blood cells per high-power field, most of which are dysmorphic) and/or cellular casts. The urine may be contaminated with vaginal blood in menstruating women or with red bladder cells with urinary tract infections. Red cells from these sources are not dysmorphic. **3).** A rising serum creatinine that is not clearly attributable to another mechanism. Our approach is consistent with the indications for kidney biopsy included in the joint European Alliance of Associations for Rheumatology (formerly known as the European League Against Rheumatism)/European Renal Association-European Dialysis and Transplant Association (EULAR/ERA-EDTA) guidelines. On the basis of clinicopathologic correlations derived from kidney biopsy, a lupus nephritis (LN) classification system was developed by a group of kidney pathologists, nephrologists, and rheumatologists in 2004 (the ISN/RPS classification) and revised in 2018 (17–19). The revised ISN/RPS classification system divides glomerular disorders associated with SLE into six different patterns (or classes) on the basis of kidney biopsy histopathology (17–19).

After studies lacking detailed information were excluded, 56 clinical trials were identified, indicating a relative scarcity of CAR-T clinical trials for ARDs. The details of these clinical trials, including the generic drug name, drug names, targets, source of CAR-T cell therapy, summary, latest change, sponsor, status, phase, drug, disease, initial date, enrollment, interventions, locations, drug country, region, drug country, mechanism of action, development status, therapeutic class, drug type, target family, and drug disease group, are compiled and included in **Supplementary Table S1**.

## 3 Results

**Figure 1:**
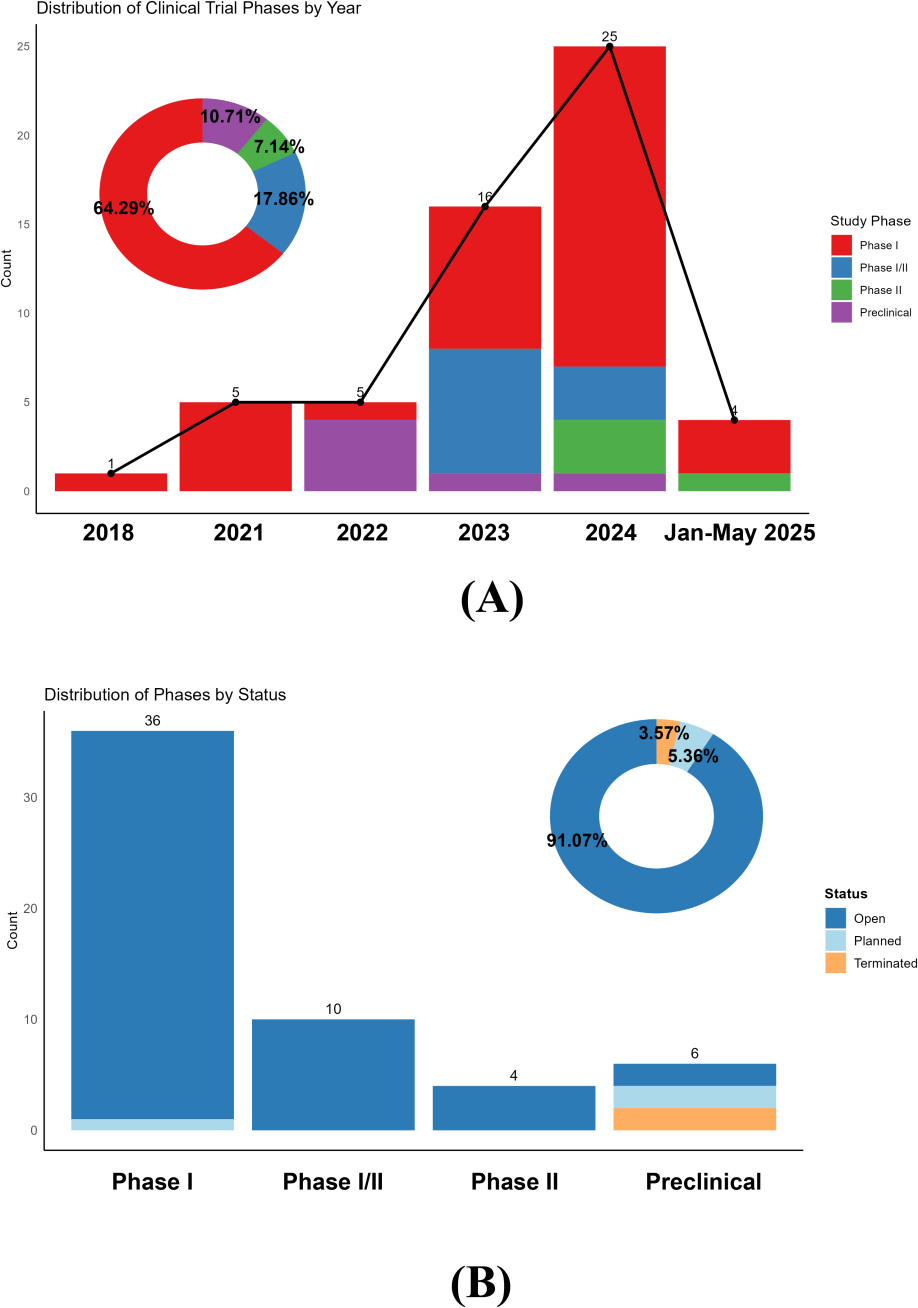
**(A)** Distribution of clinical trial phases by year. The bar chart illustrates the number of clinical trials initiated each year, categorized by phase. Note that the data for the year 2025 are incomplete and include trials registered only from January to May; it does not represent a full year’s data or a projection. The donut chart summarizes the phase distribution across all years. **(B)** Clinical phases of chimeric antigen receptor T-cell immunotherapy for autoimmune rheumatic disease clinical trials.

In 2018, a research team from Shanghai, China, registered the first clinical trial (NCT03030976) investigating CAR-T-cell therapy (anti-CD19 CAR-T cells^®^) for refractory systemic lupus erythematosus (SLE). However, the COVID-19 pandemic created a hiatus in clinical trial activity during 2019-2020, with no new CAR-T trials registered for ARDs until 2021, when five trials were initiated. Since 2021, the number of CAR-T trials targeting ARDs has steadily rebounded, peaking at 25 trials in 2024 (**Figure 1A**). The data for the year 2024 reflect a full calendar year, whereas the data for 2025 represent partial-year projections (January–May). These data reflect growing research interest in CAR-T cell therapy for ARDs, with increasing engagement from academic and industrial teams to explore its therapeutic potential.

**Figure 1A** illustrates the distribution of clinical trial phases, with phase I trials dominating (64.29%) [36/56 trials], whereas phase II trials accounted for only 7.14% (4/56 trials). This finding indicates that CAR-T cell research in ARDs remains in the early exploratory stages, primarily focused on safety, tolerability, and preliminary efficacy assessments. Notably, the marked increase in trial numbers and the increasing proportion of phase I/II and phase II trials since 2023 suggest a gradual transition toward more advanced clinical validation. Notably, the 2025 data reflect partial-year records (January-May) and are not representative of a full calendar year. The accumulated safety and efficacy data from early-phase studies may now support progression to later stages, accelerating the translation of CAR-T cell therapies into clinical practice.

**Figure 1B** details the status of ongoing trials, with ‘open’ trials comprising 91.07% (51/56 trials) of all registered studies. Most phase I, I/II, and II trials are actively recruiting or underway, reflecting robust research momentum. Only 5.36% (3/56 trials) of the trials were in the ‘planned’ stage, predominantly within preclinical or phase I settings. Terminated trials are rare (3.57%) [2/56 trials] and exclusively limited to preclinical studies, highlighting the challenges and attrition risks inherent in early-stage research.

**Figure 2:**
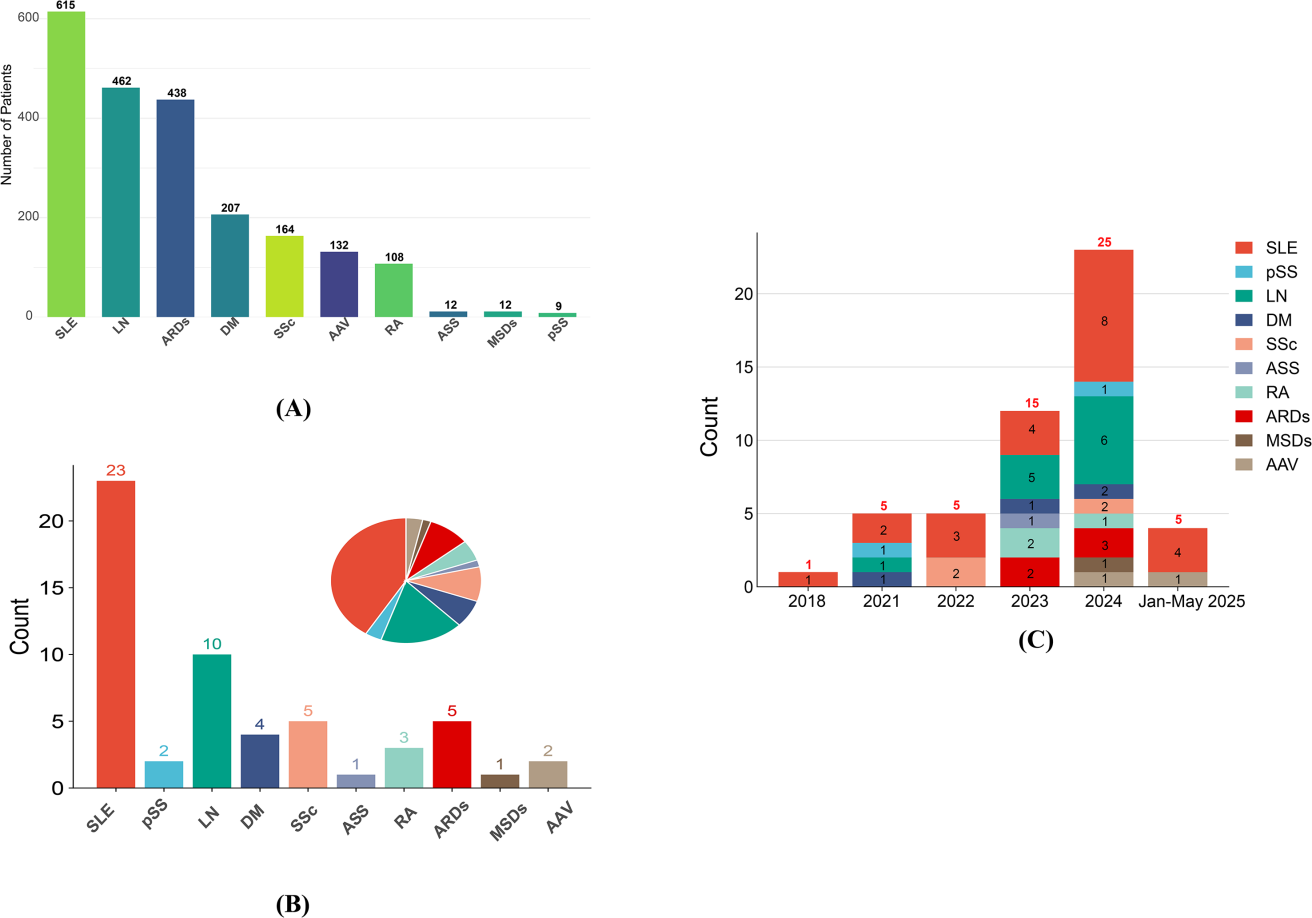
**(A)** Enrollment for different ARDs; **(B)** ARDs distribution in clinical trials; **(C)** timeline of clinical trials for various autoimmune rheumatic diseases. SLE, Systemic lupus erythematosus; pSS, primary Sjögren syndrome; LN, Lupus nephritis; DM, Dermatomyositis; SSc, Systemic sclerosis; ASS, Anti-synthetase syndrome; RA, Rheumatoid arthritis; ARDs, Autoimmune rheumatic diseases; MSDs, Musculoskeletal disorders; AAV, ANCA-associated vasculitis.

**Figure 2A** compares patient enrollment across ARDs. SLE dominated both metrics, with 615 participants (**Figure 2B**). LN, a common SLE complication, ranked second (462 participants). In contrast, pSS has minimal representation (only 9 participants). These disparities underscore the current imbalance in CAR-T-cell research focus across ARDs, with SLE and LN receiving more attention compared with other diseases.

**Figure 2C** shows the evolving landscape of trial registrations from 2018 to Jan-May 2025. The number of SLE trials has increased annually, reaching a peak in 2024. This trajectory paralleled that of the LN trials, although the LN trajectory fluctuated more. Trials for other ARDs remained sparse (0–2 annually) but showed a notable uptick in planned studies by 2024, suggesting gradual expansion of CAR-T cell research to broader ARDs subtypes. The Jan-May 2025 data suggest stabilization after the 2024 peak, potentially indicating a shift toward subtype diversification, as evidenced by new AAV and ongoing SLE trials.

**Figure 3:**
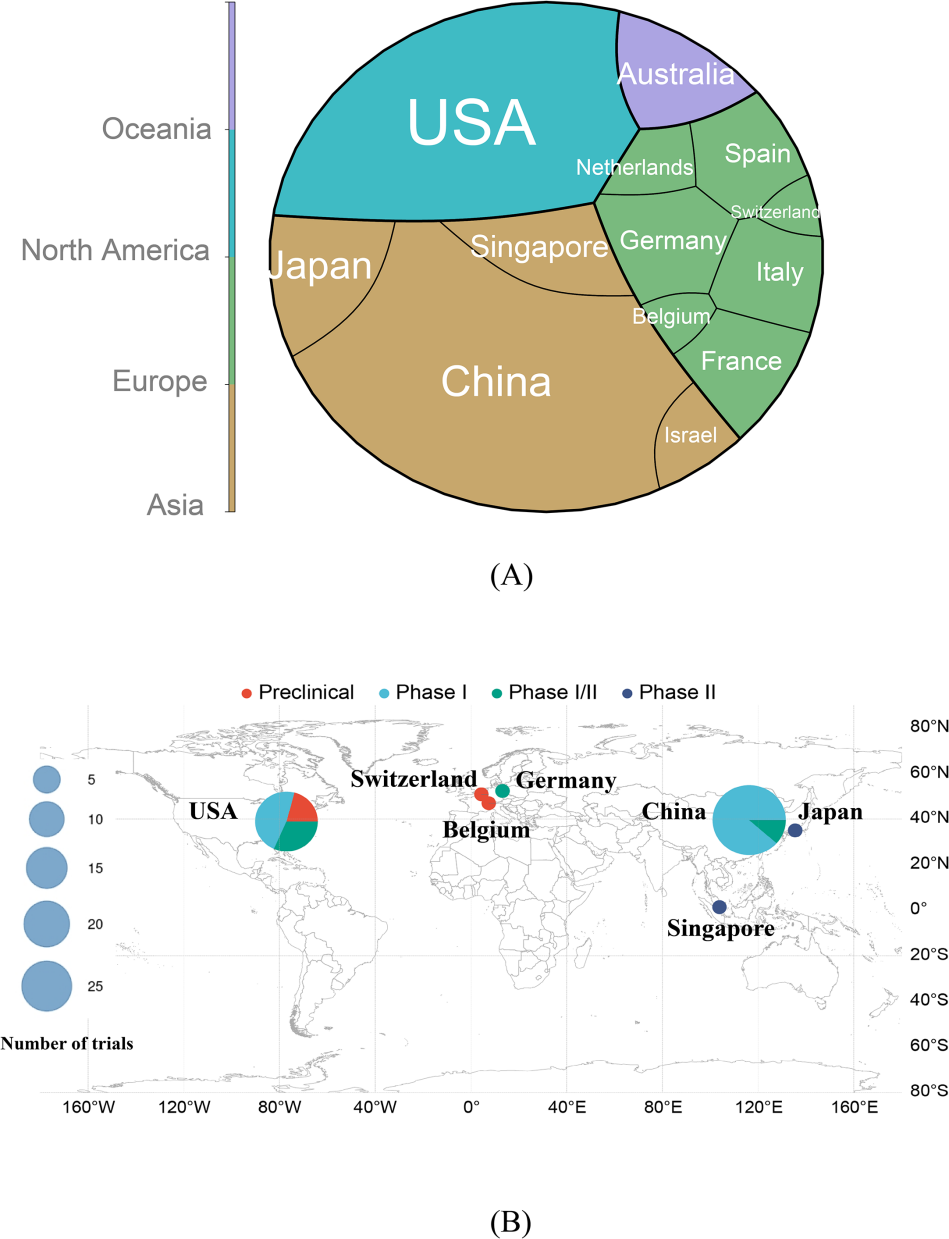
**(A)** Distribution of countries and their respective continents in chimeric antigen receptor T-cell immunotherapy clinical trials for ARDs. **(B)** Geographic distribution of countries engaged in chimeric antigen receptor T-cell immunotherapy research for autoimmune rheumatic diseases. The size of each circle corresponds to the total number of trials conducted in that country, with the scale shown in the legend. The clinical trial phases are color-coded as follows: red (Preclinical), sky blue (Phase I), green (Phase I/II), and dark blue (Phase II). Key countries, including the United States (USA), Switzerland, Germany, Belgium, China, Japan, and Singapore, are annotated to illustrate their respective research focus and trial phase distribution. This visualization highlights both the global engagement and phase-specific research efforts across different geographical regions. ARDs: Autoimmune Rheumatic Diseases.

Geospatial analysis (**Supplementary Figure S1**, **Figure 3**) highlights China and the United States as global leaders in CAR-T trials for ARDs, contributing 48.0% (27/56 trials) and 34% (19/56 trials) of the studies, respectively. China’s prominence likely stems from its large patient population, robust clinical infrastructure, and governmental support for biotechnological innovation. The U.S. leverages its oncology CAR-T cell expertise to extend to autoimmune indications. Only 2 out of 56 projects (3.6%) included both the U.S. and China. These projects are the most globally diverse (10+ countries each), suggesting that US-China cooperation occurs only in large international consortia, not bilateral projects. From a global perspective, research on CAR-T-cell therapy for ARDs is predominantly led by individual countries, which account for 83.9% (47/56 trials) of all studies. In contrast, small-scale multinational collaborations, such as the joint initiative between Japan and Singapore, are limited to only two trials. Furthermore, large-scale global cooperative research efforts are exceedingly rare, with only four such trials identified. This distribution underscores the significant collaborative barriers that exist within the international research community in this field. European contributions, while notable, are distributed across multiple countries (e.g., Germany, Spain, France, Italy), and the trial network illustrates strong intra-European collaboration, although cross-continental partnerships remain limited. Moreover, some European countries, including Switzerland and Belgium, appear only in isolated single-country projects, indicating that there is no global outreach. No trials have been registered in South America or Africa, reflecting stark global inequities in CAR-T cell research resource allocation (**Figure 3A**).

**Figure 3B** stratifies trial phases by country. The U.S. demonstrates diversified phase engagement, whereas China focuses predominantly on Phase I. Japan and Singapore, despite fewer trials, have advanced ANCA-associated vasculitis studies to Phase II. Switzerland and Belgium contributed preclinical or early-phase investigations.

**Figure 4:**
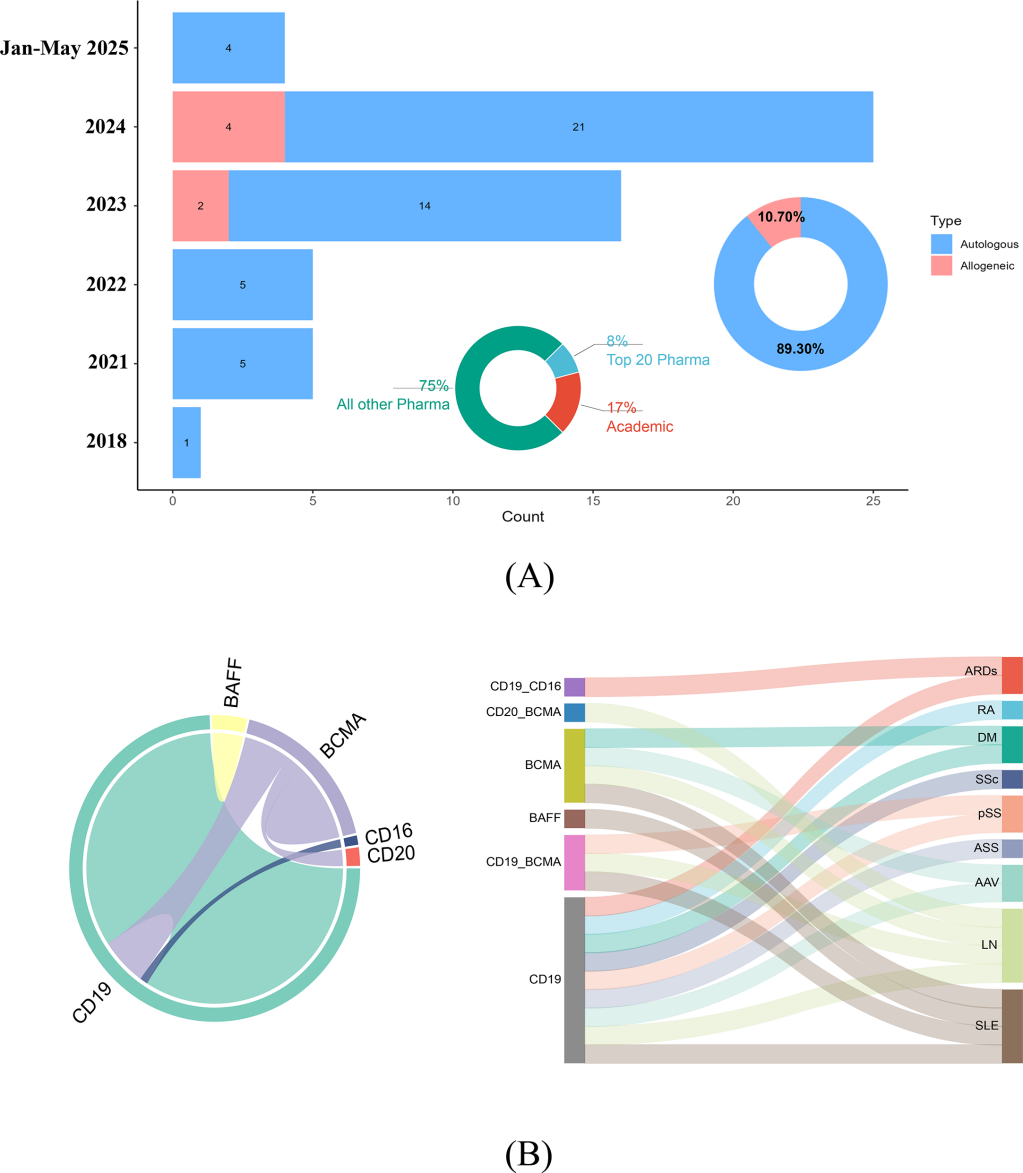
**(A)** A smaller pie chart depicts the sectoral distribution of chimeric antigen receptor T-cell studies between the pharmaceutical industry and academic institutions, highlighting their respective contributions. The larger pie chart illustrates the proportional allocation of allogeneic versus autologous chimeric antigen–receptor T-cell therapeutic approaches. The bar graph shows a marked surge in autologous chimeric antigen receptor T-cell therapeutic research from 2021-2024, with a peak of 25 clinical studies recorded in 2024. Data for the period from January to May 2025, representing ongoing or initial year-to-date figures, are also included and include 4 autologous studies. Concurrently, allogeneic CAR-T-cell studies have maintained a consistent proportion, reflecting sustained interest in this modality. **(B)** The chord diagram (left section) provides a circular layout that visually connects genetic markers and cell targets on the basis of their co-occurrence in research studies. The thickness and color intensity of the chords reflect the frequency and strength of these associations. The Sankey diagram (right section) illustrates the flow and specificity of genetic markers (e.g., BAFF, BCMA, CD19, and CD20) and cell targets across various autoimmune rheumatic diseases. The varying widths of the bands indicate the relative emphasis on specific markers and targets within each disease category. SLE, Systemic lupus erythematosus; pSS, primary Sjögren syndrome; LN, Lupus nephritis; DM, Dermatomyositis; SSc, Systemic sclerosis; ASS, Anti-synthetase syndrome; RA, Rheumatoid arthritis; ARDs, Autoimmune rheumatic diseases; AAV, ANCA-associated vasculitis.

Funding analysis (**Figure 4A**) revealed that non-leading pharmaceutical firms served as the primary sponsors of chimeric antigen receptor T-cell studies, contributing to 75% (42/56 trials) of funding entities, followed by academic institutions at 17% (10/56 trials). The top 20 pharmaceutical companies (e.g., Novartis, Galapagos NV) accounted for only 8% (4/56 trials) of funding but may facilitate commercialization through established research and development pipelines. Autologous CAR-T cell therapies dominated the clinical trials, accounting for 89.3% (50/56 trials) of all studies. The number of registered autologous CAR-T trials has increased annually since 2018, reaching a peak of 21 trials in 2024. Four autologous trials were registered from January to May 2025. Allogeneic CAR-T trials emerged more recently, with 2 trials in 2023 and 4 trials in 2024.

Exploring this intriguing phenomenon, we observe that leading pharmaceutical giants predominantly position CAR-T cell therapies within the oncology sector. As noted in the 2023 CAR-T cell therapy industry map by Tsinghua University and the National Institute of Financial Research (https://www.pbcsf.tsinghua.edu.cn/info/1090/6615.htm), the global CAR-T cell market reached an annual scale of $3 billion, with over 90% of revenue derived from hematologic malignancies. By 2025, the FDA had approved six CAR-T cell products, primarily targeting CD19^+^ B-cell malignancies (such as diffuse large B-cell lymphoma and acute lymphoblastic leukemia) and BCMA^+^ multiple myeloma (20).

In contrast, target selection for ARDs lacks uniformity, and efficacy endpoints in ARD research are more subjective, often lacking objective indicators such as overall survival (OS) or the objective response rate (ORR), which are commonly used in oncology. For example, the Systemic Lupus Erythematosus Disease Activity Index (SLEDAI), which is frequently employed to assess lupus disease activity, relies partly on physicians’ subjective judgments and patients’ self-reports. These factors contribute to the absence of any approved CAR-T cell indications for ARDs globally.

Owing to their more mature and stable returns, major pharmaceutical companies tend to invest in CAR-T cell programs within oncology. Avoiding early regulatory uncertainties and awaiting technological standardization are common strategies for these firms. This may explain why leading pharmaceutical companies have a limited presence in clinical trial investments for ARD-focused CAR-T cell therapies, maintaining a strategic wait-and-see approach.

Compared with patients with hematologic malignancies, patients with ARDs can often achieve adequate disease control through conventional treatments such as disease-modifying antirheumatic drugs (DMARDs). For example, conventional therapies (e.g., hydroxychloroquine, costing < US$1,100 annually) effectively control disease activity in more than 80% of patients with mild-to-moderate systemic lupus erythematosus (SLE), making the marginal benefit of CAR-T-cell therapy economically unjustifiable for most patients. Moreover, a 10-year trial involving 267 SLE patients demonstrated that SLEDAI scores never exceeded baseline or enrollment levels (21). Consequently, only a small subset of patients with refractory ARD represents a suitable population for CAR-T cell therapy. Additionally, research by Lungova et al. (22) highlighted that the population of patients with severe ARDs who can tolerate CAR-T cell therapy without significant organ damage may be surprisingly limited. Given this small target population and an unclear business return model, major pharmaceutical companies tend to prioritize resources for higher-volume indications such as diffuse large B-cell lymphoma and acute lymphoblastic leukemia.

Currently, autologous CAR-T cell therapies dominate the clinical landscape (accounting for 89.30% [50/56 trials]) (**Figure 4A**). This means that technical personnel must individually process cells from each patient to achieve personalized manufacturing. The associated procedures, including the programming, expansion, and induction of cells, along with the necessary medical interventions, are estimated to cost over US$500,000, significantly increasing R&D expenses for leading pharmaceutical companies (23). Consequently, the autologous CAR-T cell manufacturing model fails to leverage the GMP scale advantages of large firms and instead raises both labor costs and batch-to-batch quality control expenditures (24). In contrast, small biotech companies are more agile and can collaborate with academic centers for small-batch production, making them better suited for the development of autologous CAR-T cell therapies.

Notably, the U.S. Food and Drug Administration (FDA) recently added a black box warning to BCMA-directed and CD19-directed autologous CAR-T cell immunotherapies following observations of increased incidence of T-cell malignancies (25). Given that patients with ARDs have a significantly longer life expectancy than cancer patients do, the long-term carcinogenic risk associated with CAR-T cell therapy may be of greater concern, potentially leading major pharmaceutical companies to adopt a more cautious approach toward developing CAR-T cell therapies for ARDs.

Furthermore, several nonleading pharmaceutical companies in China, in collaboration with academic institutions, have developed CAR-T cell products that have been partially covered by medical insurance in certain cities. Consequently, these companies may bypass the global pricing strategies of larger firms and capture greater market share within these regions, supported by local reimbursement and regional healthcare policies.

Hence, the current limited investment by major pharmaceutical companies may represent a rational strategy on the basis of comprehensive risk-benefit analysis. However, with the anticipated approval of the first ARD indication, the validation of allogeneic CAR-T cell therapies for scalable manufacturing, and the full implementation of CAR-T cell insurance payment pilots, leading companies are expected to rapidly enter the market through mergers and collaborations, thereby reshaping the competitive landscape.

**Figure 4B** (chord diagram) maps therapeutic targets and disease associations. CD19 is the most frequent single target and is central to SLE management. Activated B cells in SLE drive pathogenesis via autoantigen presentation and proinflammatory cytokine secretion. CD19 CAR-T cells eliminate B cells, curbing autoantibody production while sparing long-lived plasma cells to mitigate infection risk. However, CD19 CAR-T cells fail to deplete certain autoreactive plasma cells (e.g., anti-Ro52/Ro60 producers) (26, 27). BCMA, which is highly expressed on plasma cells, has emerged as the second major target. BCMA-CAR-T cells effectively reduce autoantibodies (e.g., anti-SSA/SSB in Sjögren’s syndrome) but induce hypogammaglobulinemia, which is manageable via intravenous immunoglobulin (IVIG) supplementation. Notably, BCMA-CAR-T cells deplete hepatitis B surface antibodies (HBsAbs), necessitating revaccination post-B-cell reconstitution (27).

Dual-target CD19/BCMA-CAR-T strategies, which have been predominantly tested in SLE and LN patients, synergistically eliminate B cells and plasma cells, suppressing anti-dsDNA and anti-Ro antibodies. This approach mitigates the risk of antigen escape. Emerging targets [B-cell Activating Factor (BAFF), CD20, and CD16] remain understudied, warranting further clinical validation.

The Sankey diagram in **Figure 4B** visualizes target-disease linkages, with CD19 strongly associated with SLE. These findings underscore the evolving complexity of CAR-T cell target selection and its implications for precision immunotherapy in ARDs.

## 4 Discussion

In this study, only seven clinical trials (**Figure 4A**) utilized allogeneic CAR-T-cell therapy, while the majority (89.30% [50/56 trials]) employed conventional autologous CAR-T cell approaches (**Figure 4A**). A prior systematic review by Kattamuri et al. (28) evaluated autologous CAR-T cell outcomes in 80 ARDs patients across 24 studies. The results demonstrated that 84% of the SLE patients achieved a systemic lupus erythematosus disease activity index (SLEDAI) score of 0 at the 6-month follow-up, with 95% achieving drug-free remission. Similarly, systemic sclerosis (SSc) patients presented a mean reduction of 9 points in the modified Rodnan skin score, dermatomyositis patients presented normalized creatine kinase (CK) levels, and rheumatoid arthritis (RA) patients achieved complete remission. Notably, all patients discontinued immunosuppressive therapy by the final follow-up. Recent data further indicate sustained remission for up to 29 months in SLE patients treated with autologous CD19 CAR-T cells (14). Long-term remission following CAR-T cell therapy is closely associated with hypothesized pathways such as immune reset, T-cell memory, and durable B-cell aplasia.

**(1)** Immune reprogramming/resetting is considered the central hypothesis underlying sustained remission after CAR-T cell therapy. Through lymphodepleting chemotherapy (e.g., fludarabine + cyclophosphamide) and strong antigen stimulation (e.g., CD19^+^ B cells), the infused CAR-T cells become highly activated and expand. This process may profoundly reshape the host immune environment. The immune reprogramming/reset hypothesis proposes that CAR-T-cell therapy not only eliminates pathogenic cells but also, more importantly, “resets” the dysregulated immune system, restoring it to a more naïve and tolerant state. Using flow cytometry, Wang et al. (29) demonstrated that following TyU19 CAR-T cell therapy, patients with myositis and systemic sclerosis experienced profound B-cell depletion (complete within two weeks), with gradual recovery observed after 3–6 months. The persistent disappearance of autoantibodies (such as anti-SRP and anti-Scl-70) suggests the selective clearance of memory B cells, particularly autoreactive subsets, while naïve B cells become predominant. Concurrently, the disappearance of CD19^+^CD27^+^ plasmablasts posttreatment further supports the “rejuvenation” of the B-cell repertoire.

In a study by Mackensen A et al. (30) utilizing anti-CD19 CAR-T-cell therapy for refractory systemic lupus erythematosus, immune reset of B cells was observed approximately 100 days after infusion. Analysis of these B cells revealed nearly complete depletion of memory B cells and plasmablasts, whereas newly emerging B cells in circulation exhibited a preclass-switched naïve B-cell phenotype. All patients maintained treatment-free remission and ceased producing double-stranded DNA autoantibodies.

Furthermore, a recent study on CD19 CAR-T-cell therapy for ARDs reported the observation of B-cell immune reset at a median of 112 days, with remission sustained for up to 29 months (14). Notably, autoantibodies became undetectable, and the reconstituted B cells predominantly exhibited a naïve phenotype (14). These findings suggest that CAR-T-cell therapy may induce profound immune reset rather than simple B-cell depletion.

CAR-T cell therapy achieves deep immune remodeling through targeted elimination of pathogenic B cells, including autoreactive memory B cells and antibody-secreting plasmablasts. This process removes the source of persistent antigen presentation, creating a critical “antigen-free” window for the immune system. During subsequent reconstitution of the B-cell compartment, the emerging B-cell population demonstrates a notable shift in biological characteristics: it becomes predominantly composed of naïve, nonclass-switched B cells (characterized as IgM^+^IgD^+^), whereas previously pathogenic memory B cells and plasmablasts remain at low levels. This selective reconstitution pattern not only reduces the risk of regenerating autoreactive B cells but also supports long-term remission in ARDs by sustaining a state of immune tolerance.

**(2)** The hypothesis of induced persistent B-cell aplasia proposes that although peripheral B-cell counts may recover after CAR-T cell therapy, the administered CAR-T cells can persistently suppress B-cell lineage generation in the bone marrow or disrupt the microenvironment essential for B-cell development (such as lymphoid follicles), thereby preventing the proper maturation of new B cells and the production of autoantibodies.

Tur et al. (31) compared the efficacy of rituximab and CD19 CAR-T cell therapy in depleting tissue-resident B cells in ARDs. The results demonstrated that while rituximab clears only peripheral B cells, CAR-T cells effectively infiltrate the lymph nodes, spleen, and other lymphoid tissues, leading to the complete elimination of CD19^+^ B cells and plasma cells and even resulting in the dissolution of follicular structures and the follicular dendritic cell (FDC) network. Furthermore, Tur et al. (31) reported the absence of B cells in samples from the colon, kidney, and gallbladder following CD19 CAR-T cell therapy, whereas T cells and macrophages remained present. These findings indicate that CD19 CAR-T cell therapy is effective not only in lymphoid but also in nonlymphoid tissues, achieving comprehensive B-cell clearance. Therefore, this capacity to induce sustained B-cell aplasia and profound tissue-based B-cell depletion may represent a key mechanism underlying long-term treatment-free remission in rheumatologic patients receiving CD19 CAR-T-cell therapy.

**(3)** Following rapid *in vivo* expansion, CAR-T cells enter a contraction phase, with peripheral blood CAR-T cell counts gradually declining or becoming undetectable in some patients (32). Despite this, sustained clinical remission is observed, suggesting that CAR-T cells provide durable immune surveillance.

In hematologic malignancies, multiple studies have confirmed that CAR-T cell proliferation, efficacy, and persistence depend on the proportion of early memory or stem cell memory (SCM) T cells (33–36).

In chronic lymphocytic leukemia (CLL) patients treated with BBz CAR-T cell therapy, responders presented increased frequencies of early memory CD8^+^ T cells in their apheresis products, and these patients demonstrated significantly increased CAR-T cell expansion postinfusion (37). In contrast, nonresponders presented more effector T cells associated with exhaustion and apoptotic features, along with markedly reduced CAR-T cell expansion. Although CAR-T_SCM_ cells constitute a small fraction of the manufactured product, they rapidly expand after infusion and contribute most significantly to the clonal pool in patients with persistent CAR-T cells. Therefore, early memory CD8^+^ T cells and CAR-T_SCM_ play crucial roles in achieving long-term clinical remission in patients with hematologic malignancies by maintaining self-renewal capacity, ensuring optimal functionality, and providing durable antitumor immune responses (37).

Long-term follow-up studies in CLL patients revealed dynamic changes in CAR-T cell subsets over time, with a transition from initial CD8^+^ cytotoxic T cell dominance to later CD4^+^ helper T cell predominance (38). Notably, two CLL patients (38) maintained 97%-99% of their CD4^+^ CAR-T cell population years after treatment, with these persistent CD4^+^ CAR-T cells retaining functional activity, continuous activation markers, and proliferative capacity. Thus, the long-term efficacy of CAR-T cell therapy likely results from multiple interacting factors.

Despite these promising outcomes, autologous CAR-T cell therapy faces critical challenges in clinical implementation (28). First, ARDs patients often exhibit compromised T-cell functionality due to prior glucocorticoid and immunosuppressive therapies, complicating the isolation and expansion of sufficient functional T cells for CAR-T cell manufacturing. Moreover, autoreactive T-cell clones intrinsic to ARDs pathogenesis may inadvertently proliferate during the CAR-T cell production process. Second, the personalized nature of autologous CAR-T cell therapy incurs exorbitant costs, with direct treatment expenses exceeding approximately $165,000 USD per cycle. Additional expenses for hospitalization (≥3 weeks), intensive monitoring, and the management of therapy-related complications further exacerbate financial burdens, rendering treatment inaccessible to many ARDs patients (39, 40). Furthermore, the multistep autologous CAR-T workflow, which involves leukapheresis, genetic modification, ex vivo expansion, and reinfusion, requires meticulous interdepartmental coordination and spans several weeks (41). This protracted timeline poses significant challenges for patients with severe, refractory ARDs who have exhausted conventional therapies (e.g., immunosuppressants, biologics) and urgently require rapid intervention to mitigate end–organ damage.

In light of these challenges, allogeneic CAR-T-cell therapy has garnered increasing attention because of its abbreviated manufacturing duration (≤3 days), potential for batch production, and reduced costs (per-treatment cost below $68,000). A pioneering clinical study by a Shanghai-based team reported the first global application of universal CAR-T cells (TyUCell^®^) in treating autoimmune rheumatic diseases (ARDs) (29). Three patients with refractory ARDs (myositis, systemic sclerosis) achieved rapid clinical improvement after a single infusion: the myositis patient exhibited restoration of upper limb functionality within two weeks, and the systemic sclerosis patient demonstrated marked attenuation of cutaneous and visceral fibrosis. All patients maintained durable remission (>6 months) without receiving immunosuppressive therapy. Notably, TyUCell^®^ reduced manufacturing costs by approximately 90%, offering a safer, scalable, and accessible curative therapeutic strategy.

Major pharmaceutical companies, including BioRay Laboratories, Caribou Biosciences, and CRISPR Therapeutics, are now developing off-the-shelf CAR-T platforms. However, clinical translation remains hampered by efficacy limitations. For example, allogeneic BCMA CAR-T cell therapy by Allogene resulted in a peak CAR copy number of only 10% of the autologous counterparts and a median duration of response (mDoR) of <9 months—far inferior to the 21.8-month mDoR of autologous products. Additionally, complete response rates for universal CAR-T cell therapies are consistently lower than those for autologous therapies. Thus, future research must focus on optimizing allogeneic CAR-T cell designs to overcome the dual barriers of suboptimal efficacy and accessibility while preserving cost and scalability advantages.

The clinical translation of CAR-T-cell therapies for autoimmune rheumatic diseases (ARDs) currently faces a strategic dilemma in production model selection. Small-scale academic GMP facilities (e.g., those utilized in landmark German clinical cases (14)) offer flexibility to address individualized patient needs and accelerate early-stage proof-of-concept validation. However, their limited production capacity and inconsistent quality control hinder their ability to meet the demands of large-scale clinical trials, which require high cell yields and batch-to-batch uniformity. In contrast, pharmaceutical industry-led centralized GMP production systems leverage standardized workflows and scalable infrastructure, making them better suited for allogeneic CAR-T cell development and late-phase clinical trials. These systems also benefit from robust quality assurance frameworks and regulatory alignment, which expedite product lifecycle management.

To bridge accessibility gaps, a tiered production network must be established: regional hospital-based GMP units could address urgent or complex cases, whereas national centralized production hubs ensure standardized therapeutic supply. Concurrently, leveraging pharmaceutical GMP centers’ regulatory credibility could facilitate multinational multicenter trials. This integrated approach aims to achieve a dynamic equilibrium between personalized therapy and population-level accessibility. Notably, the majority of clinical trials in this field are industry-sponsored, with limited academic-led initiatives. However, regulatory agencies must collaborate closely to enforce stringent safety standards for allogeneic CAR-T cell therapies, ensuring patient protection while fostering innovation.

The safety of CAR-T-cell therapy in autoimmune rheumatic diseases (ARDs) centers on mitigating the risks of cytokine release syndrome (CRS) and immune effector cell-associated neurotoxicity syndrome (ICANS) (42). To further explore the risk factors for CRS/ICANS. **(1) Disease activity:** Patients with high disease activity, such as those with systemic lupus erythematosus (SLE) or systemic sclerosis (SSc), may be more susceptible to inflammatory responses following CAR-T cell therapy. In the study by Scherlinger et al., all eight included SLE patients had high pretreatment disease activity, as measured by the SLEDAI-2K. Although all patients achieved remission at six months after CAR-T cell therapy, 78% (14/18 patients) developed Grade 1–2 CRS (43). Lungova et al. suggested that a subset of SSc patients may experience rapid disease progression, leading to pulmonary and cardiovascular damage, disability, or even death. Patients with high disease activity could be more suitable candidates for CAR-T cell therapy, and early intervention may be ideal. However, special attention should be given to the risk of CRS in patients receiving CAR-T cell therapy (22). In lymphoma, a high tumor burden has been significantly correlated with an increased incidence of CRS/ICANS (44). These findings suggest that high baseline disease activity in SLE, SSc, and lymphoma patients may be associated with an elevated risk of CRS after CAR-T cell therapy. High disease activity is often accompanied by a systemic inflammatory state, in which activated immune cells, such as macrophages, are more prone to release inflammatory cytokines, thereby contributing to the development of CRS. **(2) Inflammatory markers:** Scherlinger et al. (43) reported that elevated baseline levels of C-reactive protein (CRP) and interleukin-6 (IL-6) were significant predictors of CRS following CAR-T cell therapy in patients with ARDs. Furthermore, increased pretreatment levels of CRP and ferritin have also been recognized as important risk factors for CRS and ICANS in patients with lymphoid malignancies receiving CAR-T cell therapy (45). Specifically, the level of CRP, an acute-phase reactant, is elevated in response to systemic inflammation. CAR-T cell therapy itself triggers an inflammatory response; in patients with preexisting elevated inflammatory markers, T-cell activation may excessively amplify the inflammatory cascade, thereby increasing the risk and severity of CRS and ICANS. As noted by Wilhelm et al. (46), the fundamental role of autoreactive B cells in the pathogenesis of systemic lupus erythematosus (SLE) is accompanied by B-cell-dependent enhancement of type I interferon (IFN) signaling. High baseline expression of type I IFN may further intensify the risk of CRS after CAR-T-cell infusion. Therefore, in the context of CAR-T cell immunotherapy, excessive systemic inflammation may disrupt immunoregulatory mechanisms, leading to loss of immune control and subsequent development of CRS/ICANS. Early monitoring of these inflammatory markers could assist rheumatologists and oncologists in the timely identification of patients at risk for CRS or ICANS. **(3) Lymphodepletion regimens**: Prior to CAR-T-cell administration, lymphodepleting chemotherapy has become a standard procedure. The intensity of the lymphodepleting regimen influences the immune microenvironment and subsequent response to CAR-T cells. Although more intensive conditioning regimens can more effectively clear lymphocytes and create space for CAR-T-cell engraftment, they may also lead to greater immune suppression and inflammatory responses. High-dose lymphodepletion (e.g., with fludarabine and cyclophosphamide) can cause leukopenia, anemia, neutropenic fever, and opportunistic infections, thereby increasing the incidence of Grade 1–2 CRS (47). The intensity of conditioning may also affect the release of cytokines and the balance of immune regulatory factors. High-intensity preconditioning can trigger massive cytokine release, contributing to a cytokine storm that elevates the risk of CRS. Furthermore, the intensity of lymphodepletion may alter the bone marrow microenvironment, affecting the production and function of immune cells and indirectly influencing post-CAR-T cell immune responses, potentially increasing CRS risk (48). Overall, the risk of CRS/ICANS in patients with ARDs remain relatively low. However, key risk factors include high disease activity, elevated baseline inflammatory markers, and intensive lymphodepleting regimens. Evidence from rheumatology supports the adoption of management strategies derived from oncology. Future studies should focus on personalized risk prediction and preventive measures to optimize the safety profile of CAR-T cell therapy in ARDs.

A systematic review by Fizza Zulfiqar et al. (47) reported a CRS incidence of 43.6% (44/101 patients) in ARDs patients, with ICANS occurring in 1.98% (2/101 patients) of cases, which was significantly lower than that reported in oncology populations. This attenuated toxicity profile may stem from a lower target antigen density (e.g., CD19, BCMA) on pathogenic B cells/plasma cells and baseline immunosuppression (e.g., chronic glucocorticoid use), which dampens cytokine hyperactivation. However, short follow-up durations (e.g., Müller et al. (14) reported a maximum of 29 months in SLE patients) and the multiorgan involvement of ARDs patients complicate CRS/ICANS management. Previously, Franco-Fuquen et al. (49) referenced the management of CRS and ICANS following CAR-T cell therapy in cancer patients and proposed specific management guidelines for these adverse events in patients with ARDs receiving CAR-T cell therapy. (1) CRS: CRS is the most frequent immune-related adverse event associated with CAR-T-cell therapy and results from the excessive release of cytokines such as IL-6 and TNF-α. CRS management follows the grading system proposed by the American Society for Transplantation and Cellular Therapy (ASTCT), with treatment strategies tailored to severity via a stepwise approach: Grade 1 CRS is defined by the presence of fever (body temperature ≥38 °C) without hypotension or hypoxia. Management includes antipyretics and supportive intravenous fluid therapy, along with close monitoring of vital signs. For Grade 2 CRS, where fever is accompanied by hypotension or hypoxia, first-line treatment consists of an IL-6 receptor antagonist (tocilizumab) (50). The recommended dosage is 8 mg/kg (up to a maximum of 800 mg per dose), which may be repeated every 8 hours for a maximum of four doses. In cases of refractory CRS or higher-grade events (grades 3–4), tocilizumab should be administered immediately and combined with high-potency corticosteroids such as dexamethasone (10 mg every 6 hours) or methylprednisolone (1–2 mg/kg per day) to control excessive inflammation. Severe presentations involving hypotension or hypoxia may require vasopressor support and high-flow oxygen therapy or mechanical ventilation. Monitoring the levels of biomarkers, including ferritin, C-reactive protein, and interleukin-6, is recommended for early detection and evaluation of treatment response. (2) ICANS: ICANS is another frequent complication that often occurs concurrently with or following CRS. It involves symptoms of neurotoxicity, such as impaired consciousness and seizures, and may progress to severe manifestations, including status epilepticus, cerebral edema, and coma (50). The management of ICANS is also based on the grading system established by the ASTCT (51). Grade 1 ICANS involves mild symptoms that do not require specific intervention but warrant close neurological monitoring. Grade 2 ICANS is characterized by focal neurological deficits, prompting the immediate initiation of corticosteroids, such as 10 mg dexamethasone, every 6 hours. In more severe cases (grades 3-4), methylprednisolone (1–2 mg/kg per day) is preferred. Grade 3–4 ICANS involves severe neurotoxicity, including seizures and cerebral edema, which should be managed with high-dose corticosteroids (e.g., methylprednisolone 1–2 mg/kg/day). Seizures should be treated with antiepileptic drugs such as levetiracetam, whereas cerebral edema may require osmotic therapy (e.g., mannitol or hypertonic saline) (51). Importantly, unlike CRS, tocilizumab is not effective for ICANS (52). Neurological imaging (e.g., magnetic resonance imaging (MRI)) and electroencephalography (EEG) play important roles in the evaluation of severe cases.

In December 2023, the FDA issued a black box warning for BCMA/CD19-targeted CAR-T cell therapies following accumulating reports of T-cell malignancies, including CAR transgene-positive lymphomas, suggesting the malignant transformation of engineered T cells. While no CAR-T cell-associated malignancies have been reported in ARDs to date, extended follow-up and larger cohorts may reveal similar risks. Real-world studies are urgently needed to quantify the incidence of secondary malignancies in ARDs populations and establish systematic long-term surveillance protocols. Preinfusion LD chemotherapy (fludarabine/cyclophosphamide) in ARDs carries risks of transient myelosuppression and opportunistic infections. Optimal LD dosing and regimens remain undefined for ARDs patients, warranting validation through preclinical models or comparative clinical trials. Furthermore, the impact of CAR-T cell therapy on future pregnancies remains unknown because of insufficient data, necessitating dedicated studies to evaluate fertility and gestational outcomes.

### 4.1 Future perspective

1) Development of novel CAR-based therapies: Building upon the principles of CAR-T-cell therapy, next-generation strategies such as chimeric autoantibody receptor T (CAAR-T) cells are being explored to target the source of autoantibody production while minimizing off-tissue toxicity (53). For example, CAAR-T cells engineered to recognize desmoglein-3 (Dsg3) have demonstrated efficacy in eliminating Dsg3-specific B cells, ameliorating symptoms in pemphigus vulgaris (PV) (54). Additionally, U.S. research teams have engineered CAR-modified regulatory T cells (CAR-Tregs) by reducing CAR affinity, enabling precise suppression of inflammation without adverse effects, as evidenced by preliminary success in type 1 diabetes (55). These advancements highlight the potential of CAAR-T and CAR-Treg therapies to improve clinical outcomes in ARDs patients.

2) AI-Driven Optimization of CAR Constructs: Future integration of artificial intelligence (AI) models may revolutionize CAR design by predicting structural optimizations and accelerating target antigen screening, thereby improving specificity and efficacy while reducing development timelines.

3) Combination Therapies for Enhanced Efficacy and Safety: Synergistic strategies combining CAR-T cells with biologic agents (e.g., belimumab) could balance therapeutic potency with toxicity control. CAR-T cells rapidly deplete pathogenic B cells/plasma cells (e.g., CD19^+^ B cells or BCMA^+^ plasma cells), while biologics such as BAFF inhibitors block B-cell reconstitution, reducing the risk of relapse. Such combinations may accelerate autoantibody clearance (e.g., anti-dsDNA in SLE), lower CAR-T cell dosing requirements to mitigate CRS/ICANS risks, and alleviate long-term complications such as hypogammaglobulinemia (56).

4) Multidisciplinary Collaboration: Effective CAR-T cell implementation in ARDs necessitates interdisciplinary coordination among rheumatologists, nephrologists, neurologists, specialized nurses, and immunologists. Establishing multidisciplinary CAR-T cell centers will be critical for personalized treatment protocols, toxicity prevention, and comprehensive patient management.

5) Global Safety Surveillance Networks: Leveraging existing oncology-focused CAR-T toxicity monitoring frameworks, a global collaborative adverse event reporting system should be extended to ARDs. Longitudinal safety data collection is essential to refine risk–benefit assessments and inform clinical guidelines.

6) Pediatric ARDs applications: While CD19 CAR-T cells have shown promise in pediatric SLE and juvenile dermatomyositis (57), their efficacy in other childhood chronic inflammatory rheumatic diseases remains unexplored. Multidisciplinary efforts are needed to establish pediatric-specific stratification criteria and outcome measures, facilitating broader therapeutic applications.

7) Integration of psychosocial care: Long-term CAR-T cell follow-up must incorporate assessments of anxiety, depression, treatment-related stress, and multidimensional quality-of-life metrics (physical function, social roles, and emotional health). Evidence-based psychological interventions, such as cognitive-behavioral therapy (CBT) and structured peer support groups, should be integrated into clinical pathways to address the biopsychosocial model of care.

8) Challenges in Allogeneic CAR T-cells: The strategic adoption of allogeneic CAR-T cell technology represents one of the most critical breakthroughs to overcome the core limitations of autologous therapies. Its potential to substantially reduce both treatment complexity and cost is transformative. Whereas conventional autologous CAR-T cell therapy costs approximately $165,000 USD per infusion, allogeneic CAR-T-cell therapy, through standardized, large-scale production, has the potential to reduce expenses by up to 90%, reducing the cost per treatment to less than $68,000. For example, the BRL-301 product can currently fulfill over 200 doses from a single manufacturing batch, dramatically reducing the unit cost through economies of scale (29).

More importantly, allogeneic CAR-T cells circumvent the need for the patient-specific manufacturing required in autologous approaches, eliminating individual cell collection, activation, and expansion for each patient. This significantly simplifies and shortens the treatment timeline. While autologous CAR-T cells require several weeks to months from apheresis to infusion, allogeneic CAR-T cells are produced from healthy donor cells in a standardized, large-scale process, enabling “off-the-shelf” availability and reducing the treatment timeline to just a few days. This is particularly crucial for critically ill patients in urgent need of therapy.

Furthermore, the centralized production of allogeneic CAR-T cells under controlled conditions ensures consistent quality and circumvents issues related to T-cell deficiency or dysfunction in heavily pretreated patients.

Regarding safety, a Chinese clinical team reported the use of a genetically edited allogeneic CAR-T cell product (TyU19) in three patients with severe rheumatic autoimmune diseases, which demonstrated sustained efficacy beyond six months without any incidents of grade 3 cytokine release syndrome (CRS) (29).

Despite scalability advantages, allogeneic CAR-T cell-cells face unresolved risks, including limited long-term safety data and potential genotoxicity from gene-edited donor cells.

9) Precision Patient stratification: Unlike oncology patients, most ARDs patients achieve stability with DMARDs/biologics (e.g., 67% of SLE patients maintain SLEDAI scores ≤ baseline (21)), reducing the urgency of high-risk interventions.

10) Economic considerations: The central challenge confronting CAR-T cell therapy for ARDs lies in the tension between its high treatment costs and limited marginal benefits. Currently, both autologous and allogeneic CAR-T cell therapies are prohibitively expensive. For example, a single cycle of autologous CAR-T cells costs approximately $165,000 USD, whereas allogeneic CAR-T cells cost approximately $68,000. Moreover, global disparities in healthcare accessibility are likely to exacerbate this inequity, particularly in resource-limited settings.

Conventional therapies, such as hydroxychloroquine (with an annual cost of less than approximately US$1,100), effectively control disease in more than 80% of patients with mild-to-moderate systemic lupus erythematosus (SLE). This renders the marginal benefit of CAR-T cell therapy economically unjustifiable for most healthcare systems at current price points. Although early intervention with CAR-T cells could theoretically prevent organ damage, real-world clinical decision-making is heavily influenced by economic considerations. Clinicians and health care service payers typically prioritize conventional treatments until multiple lines of therapy have failed.

Notably, the indirect economic burden resulting from long-term organ damage and disability in ARDs, such as reduced productivity and ongoing care needs, has not been adequately quantified. Current health economic assessment frameworks fail to comprehensively capture the potential long-term benefits of CAR-T cell therapy.

To increase the cost-effectiveness of CAR-T cell therapy, it is imperative to develop precise patient stratification models to identify high-benefit subgroups. These patients may include patients with progressive lupus nephritis, comorbid pulmonary fibrosis, or rapidly progressive systemic sclerosis (SSc). Early identification of high-response patient subsets, optimization of the therapeutic time window, and strategic timing of immune reset could significantly improve the cost-effectiveness ratio of CAR-T cell therapy in ARDs patients, thereby facilitating more rational and targeted utilization of this advanced treatment.

### 4.2 Limitations

This study, while comprehensive, has its limitations. First, although the Trialover database integrates clinical trial data from over 60,000 sources and updates more frequently than traditional government clinical trial databases do, it still suffers from incomplete data and potential biases within the database (not all clinical trials are registered and recorded). Second, current clinical trials lack data on special populations, such as pregnant women and ethnic minorities. Finally, the primary endpoints for ARDs-related trials primarily consisted of objective measures. However, these measures often overlook subjective symptoms, such as fatigue, depression, and anxiety, which are prevalent among the majority of ARDs patients. There is also a disconnect between the amelioration of objective indicators during clinical treatment and the lack of improvement in patients’ subjective symptoms, such as fatigue and pain.

## 5 Conclusion

Overall, CAR-T-cell therapy clinical trials for ARDs have revealed great potential but remain in their early stages. Initial findings highlight significant efficacy in some ARDs, such as SLE and LN, with autologous CAR-T cell therapy showing promise in inducing long-term drug-free remission. Nevertheless, this field faces challenges such as limited clinical data, high costs, complex production, and safety risks. The expansion of clinical trials, the application of new CAR construction technologies, strengthened multidisciplinary collaboration, and the establishment of global safety monitoring networks are expected to help overcome these obstacles gradually and promote the maturation and popularization of CAR-T cell therapy for ARDs. This progress will not only enhance the quality of life for ARDs patients and reduce disease burden but also significantly advance the development of precision medicine in ARDs.

## Data Availability

The datasets presented in this study can be found in online repositories. The names of the repository/repositories and accession number(s) can be found in the article/**Supplementary Material**.

## Supplementary material

Supplementary Figure 1 This UpSet plot visualizes the distribution of clinical trials across participating countries and their collaborative networks. The orange horizontal bars (left) indicate the total number of trials per country, with China (48% **[27/56 trials]**) and the United States (34% **[19/56 trials]**) representing the most active single contributors. The blue vertical bars (right) represent the size of the trial intersections across specific country sets. The bottom dot matrix illustrates participation patterns, confirming limited cross-national cooperation beyond singular contributions. These results highlight pronounced structural and geographic disparities in CAR-T cell trial leadership and international collaboration.
