# Supplementary figures and images for: CAR-T cell therapies in autoimmune rheumatic diseases: a brief report on the clinical trial landscape, current status, and future perspectives
Source: Front Immunol. 2025 Oct 22;16:1630569. doi: 10.3389/fimmu.2025.1630569 (PMC12586181; doi:10.3389/fimmu.2025.1630569)

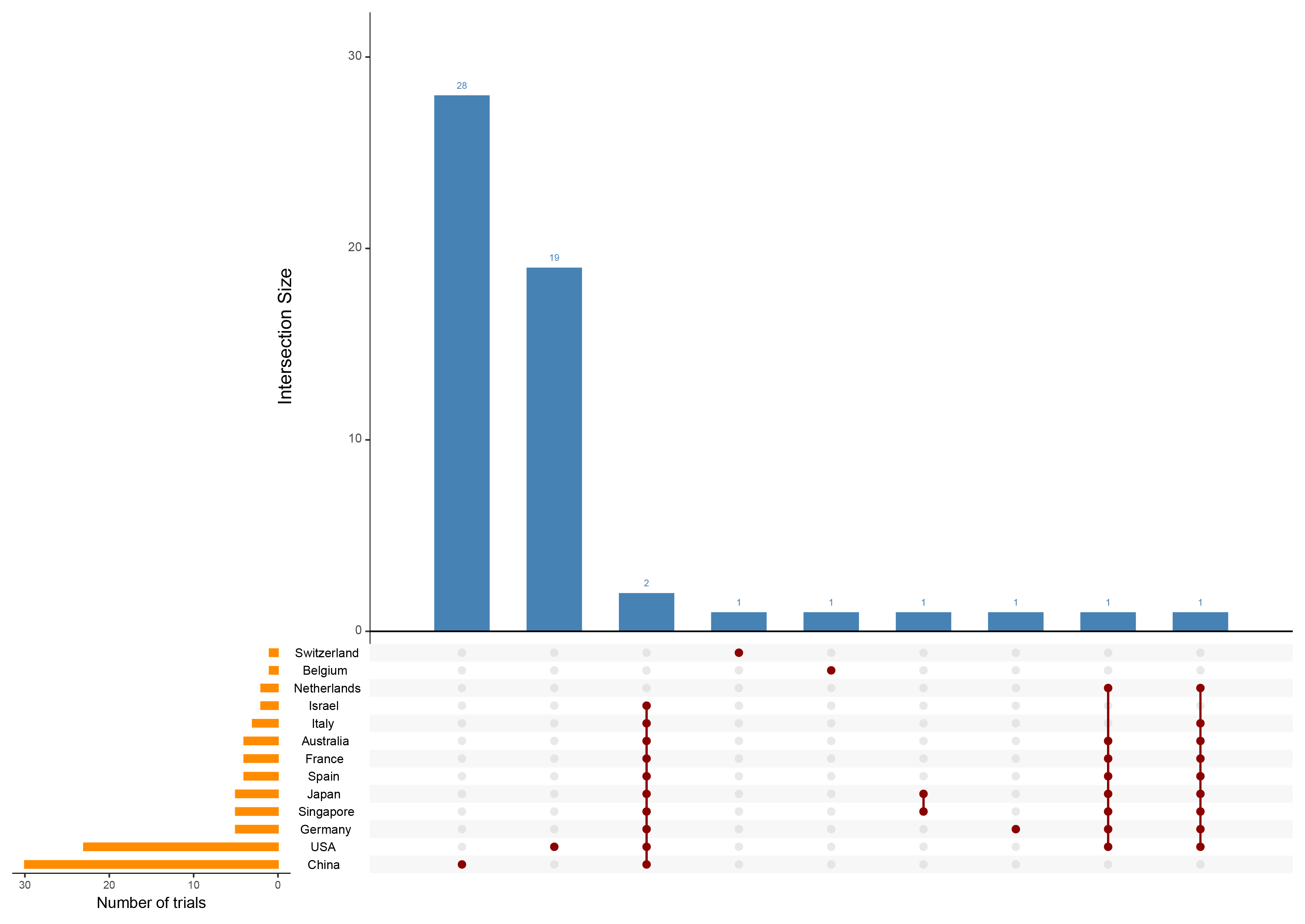

Supplement: Supplementary Figure 1 — This UpSet plot visualizes the distribution of clinical trials across participating countries and their collaborative networks. The orange horizontal bars (left) indicate the total number of trials per country, with China (48% [27/56 trials]) and the United States (34% [19/56 trials]) representing the most active single contributors. The blue vertical bars (right) represent the size of the trial intersections across specific country sets. The bottom dot matrix illustrates participation patterns, confirming limited cross-national cooperation beyond singular contributions. These results highlight pronounced structural and geographic disparities in CAR-T cell trial leadership and international collaboration. [file Image1.tif]
